# Crystal structure of hexa­kis­(μ_2_-4-*tert*-but­oxy-4-oxobut-2-en-2-olato)trizinc

**DOI:** 10.1107/S1600536814024337

**Published:** 2014-11-12

**Authors:** Olgerd O. Shtokvish, Lyudmila I. Koval, Vasyl I. Pekhnyo

**Affiliations:** a V. I. Vernadskii Institute of General and Inorganic Chemistry, Ukrainian National Academy of Sciences, Prospect Palladina 32-34, 03680 Kyiv, Ukraine

**Keywords:** crystal structure, β-keto ester, coordination compound, *tert*-butyl aceto­acetate, zinc complex

## Abstract

The title complex, systematic name hexa­kis­(μ_2_-4-*tert*-but­oxy-4-oxobut-2-en-2-olato)-1:2κ^9^ *O* ^2^,*O* ^4^:*O* ^2^;2:3κ^9^ *O* ^2^,*O* ^4^:*O* ^2^-trizinc, [Zn_3_(C_8_H_13_O_3_)_6_], syn­the­sized from *tert*-butyl aceto­acetate and di­ethyl­zinc, consists of trinuclear centrosymmetric mol­ecules of an approximate *C*_3*i*_ symmetry. The three metal cations are arranged in a linear fashion, with the central Zn^II^ atom located on a centre of symmetry. All three metal cations exhibit a distorted octa­hedral coordination geometry. The terminal Zn^II^ cations are chelated by three *tert*-butyl aceto­acetate ligands and these units are connected to the central Zn^II^ atom by the bridging enolate O atoms.

## Abstract 2

The structure of a centrosymmetric trinuclear zinc(II) complex with the formula [Zn{Zn*L*_3_}_2_], where *L* is 4-*tert*-but­oxy-4-oxobut-2-en-2-olate, is presented.

## Chemical context   

β-Dicarbonyl complexes of zinc are used to obtain ZnO films by metal-organic chemical vapour deposition (MOCVD) processes (Matthews *et al.*, 2006 ▶) and in catalysis of organic reactions (Mimoun, 2001 ▶). There are only a few reports related to the complexes of β-ketoesters with zinc and bis(ethyl aceto­acetate)­zinc(II) was described as a thermal stabil­izer for polyvinyl halide resins (Backus & Wood, 1969 ▶). Our research group has been developing coordination compounds soluble in non-polar organic solvents, including metal complexes of aceto­acetic acid esters (Koval *et al.*, 2008 ▶; Koval, Dzyuba *et al.*, 2009 ▶; Koval, Rusanov *et al.*, 2009 ▶), which can potentially be used as environmentally friendly additives for industrial products.
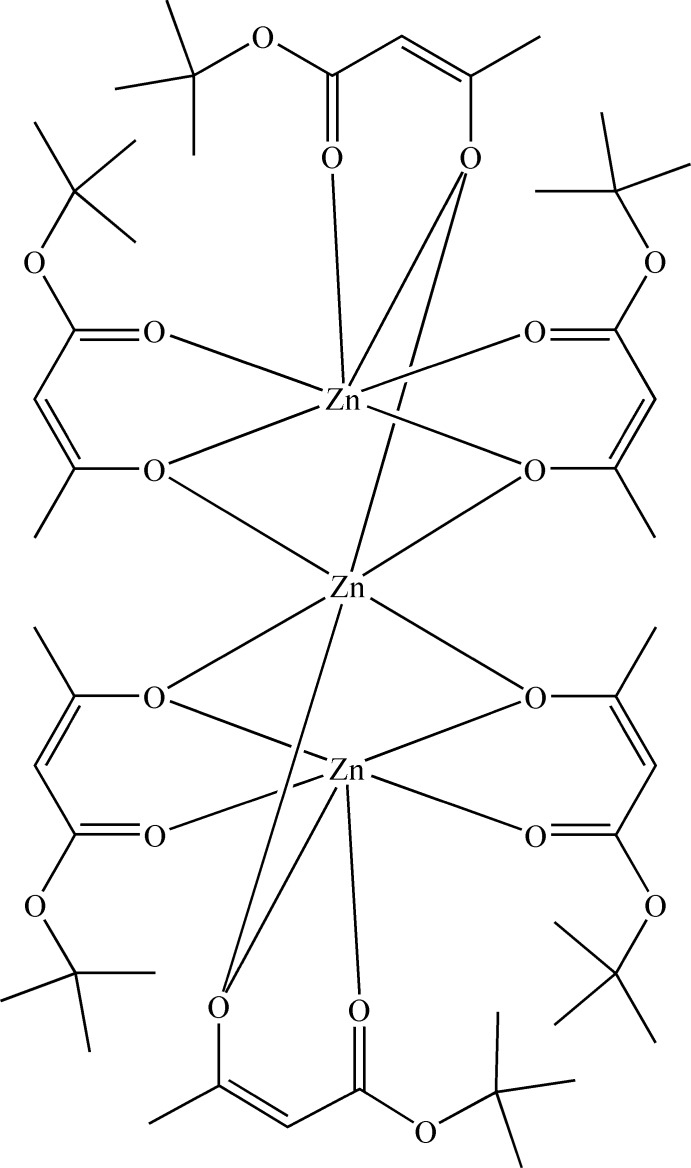


## Structural commentary   

**Figure 1:**
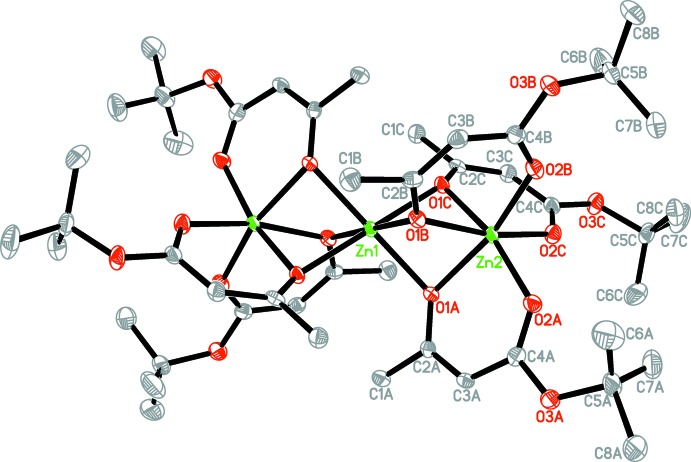
The mol­ecular structure of the title compound, showing 50% probability displacement ellipsoids. H atoms have been omitted for clarity. Unlabelled atoms are related by the symmetry operation (−*x*, 1 − *y*, −*z*).

The crystal structure of the zinc complex synthesized in our group with the formula [Zn{Zn*L*_3_}_2_], where *L* is a deproton­ated *tert*-butyl aceto­acetate ligand, is presented here (Fig. 1 ▶). In the applied labelling scheme, symmetric independence of the three ligands is reflected in the suffixes *A*, *B* and *C*, whereas the atom numbers demonstrate the complete identity of their chemical structures and mode of coordination. The mol­ecules of the title complex are trinuclear with all three zinc(II) atoms arranged in a linear fashion. The mol­ecule is centrosymmetric with atom Zn1 located on an inversion centre; however, its non-crystallographic symmetry is higher as this mol­ecule approximates *C*_3*i*_ symmetry. All Zn^II^ cations are in a distorted octa­hedral environment formed by six O atoms. Both of the symmetry-equivalent terminal Zn2 atoms are chelated through the carbonyl O2 atoms of the ester groups and the enolate O1 atoms of the aceto groups of the *tert*-butyl aceto­acetate ligands *A*, *B* and *C*. The six-membered chelate rings are virtually planar with r.m.s. deviations of 0.0257, 0.0221 and 0.0378 Å, respectively. The range of Zn2—O1 bond lengths is 2.0947 (12)–2.1160 (13) Å and these bonds are longer then Zn2—O2 bonds [2.0129 (13)–2.0365 (13) Å] (Table 1 ▶).

Two [Zn(*L*)_3_]^−^ units are connected to the central Zn1 atom *via* six bridging enolate O atoms, forming a neutral {Zn[Zn*L*_3_]_2_} mol­ecule. The crystal of this complex is related to that of the complex formed by *tert*-butyl aceto­acetate with Ni^II^ (Döhring *et al.*, 1997 ▶). Very similar complexes of Mg^II^, but with crystallographic *C*_3*i*_ symmetry, have been reported with ethyl aceto­acetate (Petrov *et al.*, 1992 ▶) and with adamantan-1-yl aceto­acetate (Koval, Dzyuba *et al.*, 2009 ▶). A common feature of these complexes is that the metal bonds to the carbonyl groups are shorter then those to the bridging enolate groups, whereas in mononuclear complexes an opposite trend has been found (Barclay & Cooper, 1965 ▶; Hall *et al.*, 1966 ▶; Fawcett *et al.*, 1997 ▶; Koval, Rusanov *et al.*, 2009 ▶). Thus, there is enough evidence to suggest that ketoesters always form {*M*[*ML*_3_]_2_} complexes with bridging enolate oxygen atoms with divalent metals with coordination number 6 when there are no other ligands able to coordinate to the central atom.

## Supra­molecular features   

**Figure 2:**
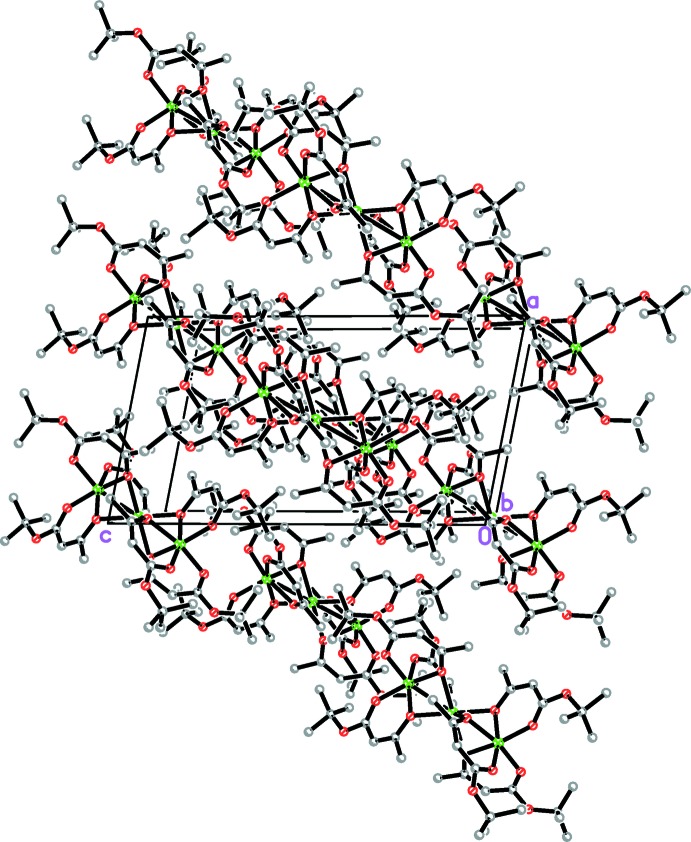
The crystal packing of the title compound, in a projection along the *b* axis. H atoms have been omitted for clarity.

**Figure 3:**
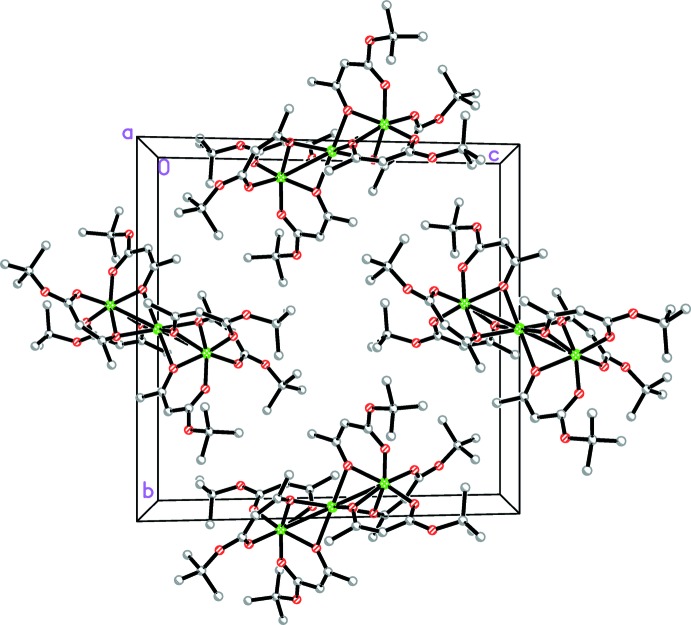
View of a mol­ecular layer in the title compound, in a projection along the *a* axis. H atoms have been omitted for clarity.

There are no short inter­molecular contacts between neighbouring mol­ecules in the crystal. The mol­ecules are closely packed into (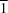
01) layers (Fig. 2 ▶). The mol­ecules within the layers are arranged so that their *tert*-butyl ends are directed towards the central parts of neighbouring mol­ecules (Fig. 3 ▶).

## Synthesis and crystallization   

To a solution of *tert*-butyl aceto­acetate (0.01 mol) in 100 ml of toluene was added dropwise 5 ml of a 1 *M* solution of Zn(C_2_H_5_)_2_ (0.005 mol) in hexane. The procedure was carried out under an argon atmosphere at 233 K with vigorous stirring. The stirring under the argon atmosphere was stopped when the cooling bath (cyclo­hexa­none with solid CO_2_) reached room temperature. Next day, the reaction mixture was evaporated and a mobile yellowish liquid was obtained. After one day, a small amount of solid hydrolysis products precipitated from the liquid. The liquid was filtered off and hexane was added. A considerable amount of precipitate was obtained. The precipitate was filtered off and washed with toluene. Crystals suitable for X-ray diffraction analysis were obtained by very slow evaporation of the solvent from the filtrate at room temperature.

## Refinement   

Crystal data, data collection and structure refinement details are summarized in Table 2 ▶. All H atoms were placed in geometrically idealized positions and constrained to ride on C atoms, with C—H bonds for the vinyl and methyl groups of 0.95 and 0.98 Å, respectively, with *U*_iso_(H_vin­yl_) = 1.2*U*_eq_(C) and *U*_iso_(H_methyl_) = 1.5*U*_eq_(C). The methyl groups were allowed to rotate freely about the C—C bonds.

## Supplementary Material

Crystal structure: contains datablock(s) I, New_Global_Publ_Block. DOI: 10.1107/S1600536814024337/gk2617sup1.cif

Structure factors: contains datablock(s) I. DOI: 10.1107/S1600536814024337/gk2617Isup2.hkl

CCDC reference: 997496

Additional supporting information:  crystallographic information; 3D view; checkCIF report

## Figures and Tables

**Table 1:** Selected bond lengths ()

O1*A*Zn1	2.0913(12)	O2*B*Zn2	2.0349(13)
O1*A*Zn2	2.1109(12)	O1*C*Zn2	2.0947(12)
O2*A*Zn2	2.0129(13)	O1*C*Zn1	2.1054(12)
O1*B*Zn1	2.0945(12)	O2*C*Zn2	2.0365(13)
O1*B*Zn2	2.1160(13)		

**Table 2:** Experimental details

Crystal data	
Chemical formula	[Zn_3_(C_8_H_13_O_3_)_6_]
*M*_r_	1139.21
Crystal system, space group	Monoclinic, *P*2_1_/*n*
Temperature (K)	100
*a*, *b*, *c* ()	9.7816(2), 16.9347(4), 17.5319(4)
()	101.096(1)
*V* (^3^)	2849.84(11)
*Z*	2
Radiation type	Mo *K*
(mm^1^)	1.32
Crystal size (mm)	0.19 0.18 0.16

Data collection	
Diffractometer	Bruker APEXII CCD
Absorption correction	Multi-scan (*SADABS*; Bruker, 2001 ▶)
*T*_min_, *T*_max_	0.792, 0.814
No. of measured, independent and observed [*I* > 2(*I*)] reflections	46427, 6684, 5163
*R*_int_	0.055
(sin /)_max_ (^1^)	0.658

Refinement	
*R*[*F* ^2^ > 2(*F* ^2^)], *wR*(*F* ^2^), *S*	0.035, 0.065, 1.01
No. of reflections	6684
No. of parameters	325
H-atom treatment	H-atom parameters constrained
_max_, _min_ (e ^3^)	0.50, 0.44

Computer programs: *APEX2* and *SAINT* (Bruker, 2007 ▶), *SHELXS97*, *SHELXL97* and *SHELXTL* (Sheldrick, 2008 ▶) and *publCIF* (Westrip, 2010 ▶).

## supplementary crystallographic information

### Crystal data

**Table:**

[Zn_3_(C_8_H_13_O_3_)_6_]	*F*(000) = 1200
*M**_r_* = 1139.21	*D*_x_ = 1.328 Mg m^−^^3^
Monoclinic, *P*2_1_/*n*	Mo *K*α radiation, λ = 0.71073 Å
Hall symbol: -P 2yn	Cell parameters from 9910 reflections
*a* = 9.7816 (2) Å	θ = 2.2–27.4°
*b* = 16.9347 (4) Å	µ = 1.32 mm^−^^1^
*c* = 17.5319 (4) Å	*T* = 100 K
β = 101.096 (1)°	Block, colourless
*V* = 2849.84 (11) Å^3^	0.19 × 0.18 × 0.16 mm
*Z* = 2	

### Data collection

**Table:**

Bruker APEXII CCD diffractometer	6684 independent reflections
Radiation source: fine-focus sealed tube	5163 reflections with *I* > 2σ(*I*)
Graphite monochromator	*R*_int_ = 0.055
φ and ω scans	θ_max_ = 27.9°, θ_min_ = 1.7°
Absorption correction: multi-scan (*SADABS*; Bruker, 2001)	*h* = −12→12
*T*_min_ = 0.792, *T*_max_ = 0.814	*k* = −22→22
46427 measured reflections	*l* = −22→22

### Refinement

**Table:**

Refinement on *F*^2^	Primary atom site location: structure-invariant direct methods
Least-squares matrix: full	Secondary atom site location: difference Fourier map
*R*[*F*^2^ > 2σ(*F*^2^)] = 0.035	Hydrogen site location: inferred from neighbouring sites
*wR*(*F*^2^) = 0.065	H-atom parameters constrained
*S* = 1.01	*w* = 1/[σ^2^(*F*_o_^2^) + (0.0222*P*)^2^ + 1.7611*P*] where *P* = (*F*_o_^2^ + 2*F*_c_^2^)/3
6684 reflections	(Δ/σ)_max_ < 0.001
325 parameters	Δρ_max_ = 0.50 e Å^−^^3^
0 restraints	Δρ_min_ = −0.44 e Å^−^^3^

### Special details

**Table:**

Geometry. All e.s.d.'s (except the e.s.d. in the dihedral angle between two l.s. planes) are estimated using the full covariance matrix. The cell e.s.d.'s are taken into account individually in the estimation of e.s.d.'s in distances, angles and torsion angles; correlations between e.s.d.'s in cell parameters are only used when they are defined by crystal symmetry. An approximate (isotropic) treatment of cell e.s.d.'s is used for estimating e.s.d.'s involving l.s. planes.
Refinement. Refinement of *F*^2^ against ALL reflections. The weighted *R*-factor *wR* and goodness of fit *S* are based on *F*^2^, conventional *R*-factors *R* are based on *F*, with *F* set to zero for negative *F*^2^. The threshold expression of *F*^2^ > σ(*F*^2^) is used only for calculating *R*-factors(gt) *etc*. and is not relevant to the choice of reflections for refinement. *R*-factors based on *F*^2^ are statistically about twice as large as those based on *F*, and *R*- factors based on ALL data will be even larger.

### Fractional atomic coordinates and isotropic or equivalent isotropic displacement parameters (Å^2^)

**Table:**

	*x*	*y*	*z*	*U*_iso_*/*U*_eq_	
C1A	−0.2103 (2)	0.39839 (11)	0.11604 (11)	0.0177 (4)	
H1A1	−0.2551	0.4094	0.0621	0.026*	
H1A2	−0.2819	0.3901	0.1474	0.026*	
H1A3	−0.1527	0.3508	0.1178	0.026*	
C2A	−0.1204 (2)	0.46710 (11)	0.14789 (10)	0.0147 (4)	
C3A	−0.1531 (2)	0.51102 (11)	0.20747 (11)	0.0165 (4)	
H3A	−0.2362	0.4981	0.2253	0.020*	
C4A	−0.0711 (2)	0.57495 (11)	0.24470 (11)	0.0177 (4)	
C5A	−0.0464 (2)	0.66654 (13)	0.35512 (13)	0.0292 (5)	
C6A	−0.0251 (3)	0.74038 (14)	0.31017 (16)	0.0435 (7)	
H6A1	0.0407	0.7293	0.2759	0.065*	
H6A2	0.0122	0.7825	0.3466	0.065*	
H6A3	−0.1145	0.7572	0.2789	0.065*	
C7A	0.0896 (3)	0.63136 (17)	0.39849 (14)	0.0413 (7)	
H7A1	0.0707	0.5808	0.4217	0.062*	
H7A2	0.1328	0.6678	0.4396	0.062*	
H7A3	0.1528	0.6228	0.3622	0.062*	
C8A	−0.1445 (3)	0.68326 (16)	0.41048 (14)	0.0411 (7)	
H8A1	−0.2339	0.7021	0.3810	0.062*	
H8A2	−0.1037	0.7237	0.4480	0.062*	
H8A3	−0.1593	0.6347	0.4382	0.062*	
O1A	−0.01240 (13)	0.47876 (7)	0.11598 (7)	0.0143 (3)	
O2A	0.03767 (14)	0.60180 (8)	0.22911 (8)	0.0189 (3)	
O3A	−0.12417 (14)	0.60575 (8)	0.30361 (8)	0.0234 (3)	
C1B	−0.1038 (2)	0.70210 (12)	−0.05633 (12)	0.0229 (5)	
H1B1	−0.1116	0.6594	−0.0947	0.034*	
H1B2	−0.0830	0.7518	−0.0804	0.034*	
H1B3	−0.1919	0.7072	−0.0380	0.034*	
C2B	0.0115 (2)	0.68343 (11)	0.01130 (11)	0.0161 (4)	
C3B	0.1147 (2)	0.73757 (11)	0.03455 (11)	0.0192 (4)	
H3B	0.1092	0.7863	0.0072	0.023*	
C4B	0.2302 (2)	0.72621 (12)	0.09693 (11)	0.0196 (4)	
C5B	0.4513 (2)	0.78613 (15)	0.16194 (13)	0.0324 (6)	
C6B	0.5420 (3)	0.71543 (17)	0.15398 (16)	0.0478 (7)	
H6B1	0.4963	0.6673	0.1674	0.072*	
H6B2	0.6323	0.7214	0.1891	0.072*	
H6B3	0.5563	0.7118	0.1003	0.072*	
C7B	0.4136 (3)	0.79200 (16)	0.24149 (13)	0.0397 (6)	
H7B1	0.3519	0.8374	0.2428	0.060*	
H7B2	0.4986	0.7989	0.2809	0.060*	
H7B3	0.3660	0.7436	0.2523	0.060*	
C8B	0.5207 (3)	0.86205 (17)	0.14291 (15)	0.0479 (7)	
H8B1	0.5404	0.8586	0.0903	0.072*	
H8B2	0.6081	0.8697	0.1803	0.072*	
H8B3	0.4584	0.9068	0.1459	0.072*	
O1B	0.00269 (13)	0.61522 (7)	0.04371 (7)	0.0155 (3)	
O2B	0.24966 (14)	0.67060 (8)	0.14360 (8)	0.0213 (3)	
O3B	0.32375 (16)	0.78496 (8)	0.10134 (8)	0.0262 (3)	
C1C	0.3522 (2)	0.42764 (12)	−0.00817 (11)	0.0202 (4)	
H1C1	0.2679	0.4021	−0.0369	0.030*	
H1C2	0.4259	0.3881	0.0059	0.030*	
H1C3	0.3830	0.4684	−0.0408	0.030*	
C2C	0.3212 (2)	0.46499 (11)	0.06449 (11)	0.0156 (4)	
C3C	0.4103 (2)	0.45240 (12)	0.13380 (11)	0.0184 (4)	
H3C	0.4914	0.4219	0.1326	0.022*	
C4C	0.3921 (2)	0.48114 (11)	0.20774 (11)	0.0173 (4)	
C5C	0.5028 (2)	0.48454 (13)	0.34655 (11)	0.0241 (5)	
C6C	0.3813 (3)	0.44889 (15)	0.37630 (13)	0.0361 (6)	
H6C1	0.2944	0.4734	0.3497	0.054*	
H6C2	0.3931	0.4582	0.4324	0.054*	
H6C3	0.3777	0.3919	0.3662	0.054*	
C7C	0.5079 (3)	0.57363 (14)	0.35287 (13)	0.0332 (6)	
H7C1	0.5825	0.5939	0.3281	0.050*	
H7C2	0.5261	0.5890	0.4078	0.050*	
H7C3	0.4185	0.5958	0.3268	0.050*	
C8C	0.6401 (3)	0.44877 (17)	0.38744 (13)	0.0414 (7)	
H8C1	0.6369	0.3913	0.3807	0.062*	
H8C2	0.6555	0.4615	0.4430	0.062*	
H8C3	0.7165	0.4705	0.3650	0.062*	
O1C	0.21038 (13)	0.50872 (7)	0.05551 (7)	0.0145 (3)	
O2C	0.29297 (14)	0.52003 (8)	0.22220 (7)	0.0190 (3)	
O3C	0.49833 (14)	0.46044 (9)	0.26487 (7)	0.0224 (3)	
Zn1	0.0000	0.5000	0.0000	0.01268 (8)	
Zn2	0.13652 (2)	0.569725 (13)	0.143328 (12)	0.01373 (6)	

### Atomic displacement parameters (Å^2^)

**Table:**

	*U*^11^	*U*^22^	*U*^33^	*U*^12^	*U*^13^	*U*^23^
C1A	0.0141 (10)	0.0179 (10)	0.0215 (10)	−0.0006 (8)	0.0048 (8)	−0.0010 (8)
C2A	0.0126 (10)	0.0164 (9)	0.0136 (9)	0.0025 (8)	−0.0009 (8)	0.0034 (8)
C3A	0.0109 (10)	0.0210 (10)	0.0181 (10)	−0.0011 (8)	0.0044 (8)	−0.0014 (8)
C4A	0.0170 (11)	0.0199 (10)	0.0161 (10)	0.0056 (8)	0.0030 (8)	0.0000 (8)
C5A	0.0246 (13)	0.0342 (13)	0.0299 (12)	−0.0067 (10)	0.0080 (10)	−0.0206 (10)
C6A	0.0477 (17)	0.0286 (13)	0.0594 (18)	−0.0070 (12)	0.0230 (14)	−0.0190 (12)
C7A	0.0283 (14)	0.0616 (17)	0.0322 (13)	−0.0047 (13)	0.0013 (11)	−0.0212 (13)
C8A	0.0341 (15)	0.0528 (16)	0.0400 (14)	−0.0087 (12)	0.0164 (12)	−0.0289 (13)
O1A	0.0111 (7)	0.0171 (7)	0.0148 (7)	−0.0019 (5)	0.0025 (5)	−0.0005 (5)
O2A	0.0153 (8)	0.0217 (7)	0.0205 (7)	−0.0023 (6)	0.0056 (6)	−0.0053 (6)
O3A	0.0185 (8)	0.0303 (8)	0.0233 (8)	−0.0032 (6)	0.0086 (6)	−0.0120 (6)
C1B	0.0229 (12)	0.0210 (10)	0.0234 (11)	0.0020 (9)	0.0008 (9)	0.0035 (9)
C2B	0.0155 (10)	0.0157 (9)	0.0189 (10)	0.0026 (8)	0.0076 (8)	−0.0005 (8)
C3B	0.0239 (12)	0.0163 (10)	0.0185 (10)	−0.0011 (8)	0.0068 (9)	0.0004 (8)
C4B	0.0213 (12)	0.0200 (10)	0.0195 (10)	−0.0048 (9)	0.0091 (9)	−0.0084 (8)
C5B	0.0260 (13)	0.0453 (14)	0.0257 (12)	−0.0196 (11)	0.0041 (10)	−0.0120 (11)
C6B	0.0231 (14)	0.0657 (19)	0.0529 (17)	−0.0094 (13)	0.0036 (12)	−0.0180 (15)
C7B	0.0401 (16)	0.0516 (16)	0.0268 (13)	−0.0213 (13)	0.0051 (11)	−0.0135 (12)
C8B	0.0465 (17)	0.0585 (18)	0.0406 (15)	−0.0358 (15)	0.0129 (13)	−0.0154 (13)
O1B	0.0140 (7)	0.0139 (6)	0.0181 (7)	−0.0001 (5)	0.0023 (6)	−0.0006 (5)
O2B	0.0201 (8)	0.0214 (7)	0.0213 (7)	−0.0061 (6)	0.0009 (6)	−0.0016 (6)
O3B	0.0282 (9)	0.0270 (8)	0.0235 (8)	−0.0154 (7)	0.0055 (7)	−0.0061 (6)
C1C	0.0163 (11)	0.0242 (10)	0.0199 (10)	0.0033 (9)	0.0029 (8)	−0.0028 (9)
C2C	0.0105 (10)	0.0150 (9)	0.0221 (10)	−0.0025 (8)	0.0051 (8)	−0.0002 (8)
C3C	0.0127 (10)	0.0225 (10)	0.0198 (10)	0.0044 (8)	0.0024 (8)	−0.0014 (8)
C4C	0.0139 (11)	0.0179 (10)	0.0189 (10)	−0.0014 (8)	−0.0001 (8)	0.0037 (8)
C5C	0.0205 (12)	0.0362 (13)	0.0136 (10)	0.0052 (9)	−0.0018 (9)	0.0002 (9)
C6C	0.0389 (15)	0.0461 (15)	0.0232 (12)	−0.0007 (12)	0.0060 (11)	0.0085 (10)
C7C	0.0307 (14)	0.0380 (13)	0.0270 (12)	−0.0034 (11)	−0.0044 (10)	−0.0067 (10)
C8C	0.0358 (15)	0.0634 (18)	0.0208 (12)	0.0200 (13)	−0.0054 (11)	0.0003 (12)
O1C	0.0100 (7)	0.0175 (7)	0.0153 (7)	0.0013 (5)	0.0008 (5)	−0.0022 (5)
O2C	0.0152 (8)	0.0240 (7)	0.0167 (7)	0.0039 (6)	0.0006 (6)	−0.0015 (6)
O3C	0.0168 (8)	0.0319 (8)	0.0161 (7)	0.0092 (6)	−0.0027 (6)	0.0001 (6)
Zn1	0.01010 (16)	0.01458 (15)	0.01283 (15)	−0.00013 (13)	0.00090 (12)	−0.00181 (12)
Zn2	0.01023 (12)	0.01621 (11)	0.01412 (11)	−0.00051 (9)	0.00074 (8)	−0.00224 (9)

### Geometric parameters (Å, º)

**Table:**

C1A—C2A	1.500 (3)	C6B—H6B3	0.9800
C1A—H1A1	0.9800	C7B—H7B1	0.9800
C1A—H1A2	0.9800	C7B—H7B2	0.9800
C1A—H1A3	0.9800	C7B—H7B3	0.9800
C2A—O1A	1.302 (2)	C8B—H8B1	0.9800
C2A—C3A	1.369 (3)	C8B—H8B2	0.9800
C3A—C4A	1.428 (3)	C8B—H8B3	0.9800
C3A—H3A	0.9500	O1B—Zn1	2.0945 (12)
C4A—O2A	1.235 (2)	O1B—Zn2	2.1160 (13)
C4A—O3A	1.347 (2)	O2B—Zn2	2.0349 (13)
C5A—O3A	1.480 (2)	C1C—C2C	1.505 (3)
C5A—C6A	1.514 (3)	C1C—H1C1	0.9800
C5A—C8A	1.518 (3)	C1C—H1C2	0.9800
C5A—C7A	1.520 (3)	C1C—H1C3	0.9800
C6A—H6A1	0.9800	C2C—O1C	1.297 (2)
C6A—H6A2	0.9800	C2C—C3C	1.369 (3)
C6A—H6A3	0.9800	C3C—C4C	1.428 (3)
C7A—H7A1	0.9800	C3C—H3C	0.9500
C7A—H7A2	0.9800	C4C—O2C	1.238 (2)
C7A—H7A3	0.9800	C4C—O3C	1.344 (2)
C8A—H8A1	0.9800	C5C—O3C	1.482 (2)
C8A—H8A2	0.9800	C5C—C6C	1.512 (3)
C8A—H8A3	0.9800	C5C—C7C	1.513 (3)
O1A—Zn1	2.0913 (12)	C5C—C8C	1.522 (3)
O1A—Zn2	2.1109 (12)	C6C—H6C1	0.9800
O2A—Zn2	2.0129 (13)	C6C—H6C2	0.9800
C1B—C2B	1.505 (3)	C6C—H6C3	0.9800
C1B—H1B1	0.9800	C7C—H7C1	0.9800
C1B—H1B2	0.9800	C7C—H7C2	0.9800
C1B—H1B3	0.9800	C7C—H7C3	0.9800
C2B—O1B	1.298 (2)	C8C—H8C1	0.9800
C2B—C3B	1.367 (3)	C8C—H8C2	0.9800
C3B—C4B	1.426 (3)	C8C—H8C3	0.9800
C3B—H3B	0.9500	O1C—Zn2	2.0947 (12)
C4B—O2B	1.238 (2)	O1C—Zn1	2.1054 (12)
C4B—O3B	1.344 (2)	O2C—Zn2	2.0365 (13)
C5B—O3B	1.475 (3)	Zn1—O1A^i^	2.0913 (12)
C5B—C7B	1.513 (3)	Zn1—O1B^i^	2.0945 (12)
C5B—C6B	1.513 (4)	Zn1—O1C^i^	2.1054 (12)
C5B—C8B	1.521 (3)	Zn1—Zn2^i^	2.8636 (2)
C6B—H6B1	0.9800	Zn1—Zn2	2.8636 (2)
C6B—H6B2	0.9800		
			
C2A—C1A—H1A1	109.5	C2C—C1C—H1C3	109.5
C2A—C1A—H1A2	109.5	H1C1—C1C—H1C3	109.5
H1A1—C1A—H1A2	109.5	H1C2—C1C—H1C3	109.5
C2A—C1A—H1A3	109.5	O1C—C2C—C3C	124.84 (18)
H1A1—C1A—H1A3	109.5	O1C—C2C—C1C	115.93 (16)
H1A2—C1A—H1A3	109.5	C3C—C2C—C1C	119.22 (17)
O1A—C2A—C3A	124.97 (17)	C2C—C3C—C4C	125.66 (18)
O1A—C2A—C1A	115.07 (16)	C2C—C3C—H3C	117.2
C3A—C2A—C1A	119.94 (18)	C4C—C3C—H3C	117.2
C2A—C3A—C4A	124.55 (18)	O2C—C4C—O3C	120.67 (17)
C2A—C3A—H3A	117.7	O2C—C4C—C3C	127.38 (18)
C4A—C3A—H3A	117.7	O3C—C4C—C3C	111.94 (17)
O2A—C4A—O3A	120.16 (18)	O3C—C5C—C6C	110.16 (17)
O2A—C4A—C3A	127.59 (18)	O3C—C5C—C7C	109.87 (17)
O3A—C4A—C3A	112.25 (17)	C6C—C5C—C7C	113.0 (2)
O3A—C5A—C6A	111.28 (18)	O3C—C5C—C8C	101.81 (16)
O3A—C5A—C8A	101.87 (17)	C6C—C5C—C8C	111.08 (19)
C6A—C5A—C8A	110.2 (2)	C7C—C5C—C8C	110.4 (2)
O3A—C5A—C7A	109.28 (18)	C5C—C6C—H6C1	109.5
C6A—C5A—C7A	112.9 (2)	C5C—C6C—H6C2	109.5
C8A—C5A—C7A	110.7 (2)	H6C1—C6C—H6C2	109.5
C5A—C6A—H6A1	109.5	C5C—C6C—H6C3	109.5
C5A—C6A—H6A2	109.5	H6C1—C6C—H6C3	109.5
H6A1—C6A—H6A2	109.5	H6C2—C6C—H6C3	109.5
C5A—C6A—H6A3	109.5	C5C—C7C—H7C1	109.5
H6A1—C6A—H6A3	109.5	C5C—C7C—H7C2	109.5
H6A2—C6A—H6A3	109.5	H7C1—C7C—H7C2	109.5
C5A—C7A—H7A1	109.5	C5C—C7C—H7C3	109.5
C5A—C7A—H7A2	109.5	H7C1—C7C—H7C3	109.5
H7A1—C7A—H7A2	109.5	H7C2—C7C—H7C3	109.5
C5A—C7A—H7A3	109.5	C5C—C8C—H8C1	109.5
H7A1—C7A—H7A3	109.5	C5C—C8C—H8C2	109.5
H7A2—C7A—H7A3	109.5	H8C1—C8C—H8C2	109.5
C5A—C8A—H8A1	109.5	C5C—C8C—H8C3	109.5
C5A—C8A—H8A2	109.5	H8C1—C8C—H8C3	109.5
H8A1—C8A—H8A2	109.5	H8C2—C8C—H8C3	109.5
C5A—C8A—H8A3	109.5	C2C—O1C—Zn2	126.21 (12)
H8A1—C8A—H8A3	109.5	C2C—O1C—Zn1	137.37 (12)
H8A2—C8A—H8A3	109.5	Zn2—O1C—Zn1	85.97 (5)
C2A—O1A—Zn1	130.34 (11)	C4C—O2C—Zn2	126.63 (12)
C2A—O1A—Zn2	126.49 (11)	C4C—O3C—C5C	121.54 (15)
Zn1—O1A—Zn2	85.91 (5)	O1A^i^—Zn1—O1A	180.00 (7)
C4A—O2A—Zn2	128.62 (13)	O1A^i^—Zn1—O1B^i^	78.67 (5)
C4A—O3A—C5A	120.56 (16)	O1A—Zn1—O1B^i^	101.33 (5)
C2B—C1B—H1B1	109.5	O1A^i^—Zn1—O1B	101.33 (5)
C2B—C1B—H1B2	109.5	O1A—Zn1—O1B	78.67 (5)
H1B1—C1B—H1B2	109.5	O1B^i^—Zn1—O1B	180.00 (7)
C2B—C1B—H1B3	109.5	O1A^i^—Zn1—O1C^i^	78.29 (5)
H1B1—C1B—H1B3	109.5	O1A—Zn1—O1C^i^	101.71 (5)
H1B2—C1B—H1B3	109.5	O1B^i^—Zn1—O1C^i^	79.82 (5)
O1B—C2B—C3B	125.05 (18)	O1B—Zn1—O1C^i^	100.18 (5)
O1B—C2B—C1B	115.42 (17)	O1A^i^—Zn1—O1C	101.71 (5)
C3B—C2B—C1B	119.53 (17)	O1A—Zn1—O1C	78.29 (5)
C2B—C3B—C4B	124.67 (18)	O1B^i^—Zn1—O1C	100.18 (5)
C2B—C3B—H3B	117.7	O1B—Zn1—O1C	79.82 (5)
C4B—C3B—H3B	117.7	O1C^i^—Zn1—O1C	180.0
O2B—C4B—O3B	120.59 (18)	O1A^i^—Zn1—Zn2^i^	47.33 (3)
O2B—C4B—C3B	127.44 (18)	O1A—Zn1—Zn2^i^	132.67 (3)
O3B—C4B—C3B	111.98 (17)	O1B^i^—Zn1—Zn2^i^	47.47 (3)
O3B—C5B—C7B	110.05 (19)	O1B—Zn1—Zn2^i^	132.53 (3)
O3B—C5B—C6B	110.66 (18)	O1C^i^—Zn1—Zn2^i^	46.86 (3)
C7B—C5B—C6B	112.9 (2)	O1C—Zn1—Zn2^i^	133.14 (3)
O3B—C5B—C8B	101.83 (19)	O1A^i^—Zn1—Zn2	132.67 (3)
C7B—C5B—C8B	110.2 (2)	O1A—Zn1—Zn2	47.33 (3)
C6B—C5B—C8B	110.7 (2)	O1B^i^—Zn1—Zn2	132.53 (3)
C5B—C6B—H6B1	109.5	O1B—Zn1—Zn2	47.47 (3)
C5B—C6B—H6B2	109.5	O1C^i^—Zn1—Zn2	133.14 (3)
H6B1—C6B—H6B2	109.5	O1C—Zn1—Zn2	46.86 (3)
C5B—C6B—H6B3	109.5	Zn2^i^—Zn1—Zn2	180.000 (5)
H6B1—C6B—H6B3	109.5	O2A—Zn2—O2B	96.37 (5)
H6B2—C6B—H6B3	109.5	O2A—Zn2—O2C	90.60 (5)
C5B—C7B—H7B1	109.5	O2B—Zn2—O2C	90.47 (6)
C5B—C7B—H7B2	109.5	O2A—Zn2—O1C	164.90 (5)
H7B1—C7B—H7B2	109.5	O2B—Zn2—O1C	98.71 (5)
C5B—C7B—H7B3	109.5	O2C—Zn2—O1C	88.56 (5)
H7B1—C7B—H7B3	109.5	O2A—Zn2—O1A	87.53 (5)
H7B2—C7B—H7B3	109.5	O2B—Zn2—O1A	164.65 (5)
C5B—C8B—H8B1	109.5	O2C—Zn2—O1A	104.37 (5)
C5B—C8B—H8B2	109.5	O1C—Zn2—O1A	78.09 (5)
H8B1—C8B—H8B2	109.5	O2A—Zn2—O1B	102.04 (5)
C5B—C8B—H8B3	109.5	O2B—Zn2—O1B	86.91 (5)
H8B1—C8B—H8B3	109.5	O2C—Zn2—O1B	167.30 (5)
H8B2—C8B—H8B3	109.5	O1C—Zn2—O1B	79.57 (5)
C2B—O1B—Zn1	131.79 (12)	O1A—Zn2—O1B	77.76 (5)
C2B—O1B—Zn2	127.07 (12)	O2A—Zn2—Zn1	123.85 (4)
Zn1—O1B—Zn2	85.70 (5)	O2B—Zn2—Zn1	120.65 (4)
C4B—O2B—Zn2	128.51 (13)	O2C—Zn2—Zn1	126.01 (4)
C4B—O3B—C5B	121.57 (17)	O1C—Zn2—Zn1	47.17 (3)
C2C—C1C—H1C1	109.5	O1A—Zn2—Zn1	46.76 (3)
C2C—C1C—H1C2	109.5	O1B—Zn2—Zn1	46.83 (3)
H1C1—C1C—H1C2	109.5		
			
O1A—C2A—C3A—C4A	−1.7 (3)	C4A—O2A—Zn2—O1A	−4.92 (16)
C1A—C2A—C3A—C4A	176.66 (18)	C4A—O2A—Zn2—O1B	72.01 (17)
C2A—C3A—C4A—O2A	1.7 (3)	C4A—O2A—Zn2—Zn1	26.37 (18)
C2A—C3A—C4A—O3A	−177.40 (18)	C4B—O2B—Zn2—O2A	−103.74 (17)
C3A—C2A—O1A—Zn1	−123.31 (18)	C4B—O2B—Zn2—O2C	165.60 (17)
C1A—C2A—O1A—Zn1	58.2 (2)	C4B—O2B—Zn2—O1C	76.99 (17)
C3A—C2A—O1A—Zn2	−2.5 (3)	C4B—O2B—Zn2—O1A	0.3 (3)
C1A—C2A—O1A—Zn2	179.04 (11)	C4B—O2B—Zn2—O1B	−1.96 (16)
O3A—C4A—O2A—Zn2	−178.06 (12)	C4B—O2B—Zn2—Zn1	32.11 (18)
C3A—C4A—O2A—Zn2	2.9 (3)	C4C—O2C—Zn2—O2A	173.95 (16)
O2A—C4A—O3A—C5A	−6.0 (3)	C4C—O2C—Zn2—O2B	−89.67 (16)
C3A—C4A—O3A—C5A	173.15 (17)	C4C—O2C—Zn2—O1C	9.03 (16)
C6A—C5A—O3A—C4A	62.1 (2)	C4C—O2C—Zn2—O1A	86.35 (16)
C8A—C5A—O3A—C4A	179.56 (18)	C4C—O2C—Zn2—O1B	−11.7 (3)
C7A—C5A—O3A—C4A	−63.3 (2)	C4C—O2C—Zn2—Zn1	39.83 (17)
O1B—C2B—C3B—C4B	1.6 (3)	C2C—O1C—Zn2—O2A	−90.9 (2)
C1B—C2B—C3B—C4B	−179.05 (19)	Zn1—O1C—Zn2—O2A	58.7 (2)
C2B—C3B—C4B—O2B	−7.0 (3)	C2C—O1C—Zn2—O2B	86.27 (14)
C2B—C3B—C4B—O3B	172.85 (18)	Zn1—O1C—Zn2—O2B	−124.14 (5)
C3B—C2B—O1B—Zn1	−120.29 (19)	C2C—O1C—Zn2—O2C	−3.98 (14)
C1B—C2B—O1B—Zn1	60.3 (2)	Zn1—O1C—Zn2—O2C	145.61 (5)
C3B—C2B—O1B—Zn2	3.0 (3)	C2C—O1C—Zn2—O1A	−108.99 (14)
C1B—C2B—O1B—Zn2	−176.35 (12)	Zn1—O1C—Zn2—O1A	40.60 (4)
O3B—C4B—O2B—Zn2	−173.15 (12)	C2C—O1C—Zn2—O1B	171.47 (15)
C3B—C4B—O2B—Zn2	6.6 (3)	Zn1—O1C—Zn2—O1B	−38.93 (4)
O2B—C4B—O3B—C5B	−0.8 (3)	C2C—O1C—Zn2—Zn1	−149.59 (16)
C3B—C4B—O3B—C5B	179.42 (17)	C2A—O1A—Zn2—O2A	4.72 (14)
C7B—C5B—O3B—C4B	−63.1 (3)	Zn1—O1A—Zn2—O2A	143.70 (5)
C6B—C5B—O3B—C4B	62.3 (3)	C2A—O1A—Zn2—O2B	−100.5 (2)
C8B—C5B—O3B—C4B	−179.99 (18)	Zn1—O1A—Zn2—O2B	38.5 (2)
O1C—C2C—C3C—C4C	3.9 (3)	C2A—O1A—Zn2—O2C	94.71 (14)
C1C—C2C—C3C—C4C	−177.37 (18)	Zn1—O1A—Zn2—O2C	−126.32 (5)
C2C—C3C—C4C—O2C	1.8 (3)	C2A—O1A—Zn2—O1C	−179.91 (15)
C2C—C3C—C4C—O3C	−178.79 (19)	Zn1—O1A—Zn2—O1C	−40.94 (4)
C3C—C2C—O1C—Zn2	−1.0 (3)	C2A—O1A—Zn2—O1B	−98.16 (14)
C1C—C2C—O1C—Zn2	−179.76 (12)	Zn1—O1A—Zn2—O1B	40.81 (4)
C3C—C2C—O1C—Zn1	−132.84 (18)	C2A—O1A—Zn2—Zn1	−138.97 (16)
C1C—C2C—O1C—Zn1	48.4 (2)	C2B—O1B—Zn2—O2A	93.17 (15)
O3C—C4C—O2C—Zn2	171.06 (12)	Zn1—O1B—Zn2—O2A	−125.50 (5)
C3C—C4C—O2C—Zn2	−9.6 (3)	C2B—O1B—Zn2—O2B	−2.68 (14)
O2C—C4C—O3C—C5C	−1.3 (3)	Zn1—O1B—Zn2—O2B	138.64 (5)
C3C—C4C—O3C—C5C	179.26 (17)	C2B—O1B—Zn2—O2C	−81.0 (3)
C6C—C5C—O3C—C4C	62.8 (2)	Zn1—O1B—Zn2—O2C	60.3 (2)
C7C—C5C—O3C—C4C	−62.3 (2)	C2B—O1B—Zn2—O1C	−102.13 (15)
C8C—C5C—O3C—C4C	−179.26 (19)	Zn1—O1B—Zn2—O1C	39.19 (5)
C2A—O1A—Zn1—O1B^i^	−84.97 (15)	C2B—O1B—Zn2—O1A	177.93 (15)
Zn2—O1A—Zn1—O1B^i^	138.85 (5)	Zn1—O1B—Zn2—O1A	−40.75 (4)
C2A—O1A—Zn1—O1B	95.03 (15)	C2B—O1B—Zn2—Zn1	−141.32 (16)
Zn2—O1A—Zn1—O1B	−41.15 (5)	O1A^i^—Zn1—Zn2—O2A	134.58 (7)
C2A—O1A—Zn1—O1C^i^	−3.17 (16)	O1A—Zn1—Zn2—O2A	−45.42 (7)
Zn2—O1A—Zn1—O1C^i^	−139.35 (4)	O1B^i^—Zn1—Zn2—O2A	−106.54 (7)
C2A—O1A—Zn1—O1C	176.83 (16)	O1B—Zn1—Zn2—O2A	73.46 (7)
Zn2—O1A—Zn1—O1C	40.65 (4)	O1C^i^—Zn1—Zn2—O2A	15.54 (7)
C2A—O1A—Zn1—Zn2^i^	−43.82 (16)	O1C—Zn1—Zn2—O2A	−164.46 (7)
Zn2—O1A—Zn1—Zn2^i^	180.0	O1A^i^—Zn1—Zn2—O2B	11.04 (7)
C2A—O1A—Zn1—Zn2	136.18 (16)	O1A—Zn1—Zn2—O2B	−168.96 (7)
C2B—O1B—Zn1—O1A^i^	−0.92 (17)	O1B^i^—Zn1—Zn2—O2B	129.92 (7)
Zn2—O1B—Zn1—O1A^i^	−138.95 (4)	O1B—Zn1—Zn2—O2B	−50.08 (7)
C2B—O1B—Zn1—O1A	179.08 (17)	O1C^i^—Zn1—Zn2—O2B	−108.01 (7)
Zn2—O1B—Zn1—O1A	41.05 (5)	O1C—Zn1—Zn2—O2B	71.99 (7)
C2B—O1B—Zn1—O1C^i^	−80.89 (16)	O1A^i^—Zn1—Zn2—O2C	−105.22 (7)
Zn2—O1B—Zn1—O1C^i^	141.08 (4)	O1A—Zn1—Zn2—O2C	74.78 (7)
C2B—O1B—Zn1—O1C	99.11 (16)	O1B^i^—Zn1—Zn2—O2C	13.66 (7)
Zn2—O1B—Zn1—O1C	−38.92 (4)	O1B—Zn1—Zn2—O2C	−166.34 (7)
C2B—O1B—Zn1—Zn2^i^	−41.97 (18)	O1C^i^—Zn1—Zn2—O2C	135.74 (7)
Zn2—O1B—Zn1—Zn2^i^	180.0	O1C—Zn1—Zn2—O2C	−44.26 (7)
C2B—O1B—Zn1—Zn2	138.03 (18)	O1A^i^—Zn1—Zn2—O1C	−60.95 (7)
C2C—O1C—Zn1—O1A^i^	−78.11 (18)	O1A—Zn1—Zn2—O1C	119.05 (7)
Zn2—O1C—Zn1—O1A^i^	138.97 (4)	O1B^i^—Zn1—Zn2—O1C	57.93 (7)
C2C—O1C—Zn1—O1A	101.89 (18)	O1B—Zn1—Zn2—O1C	−122.07 (7)
Zn2—O1C—Zn1—O1A	−41.03 (4)	O1C^i^—Zn1—Zn2—O1C	180.0
C2C—O1C—Zn1—O1B^i^	2.29 (18)	O1A^i^—Zn1—Zn2—O1A	180.0
Zn2—O1C—Zn1—O1B^i^	−140.63 (5)	O1B^i^—Zn1—Zn2—O1A	−61.12 (7)
C2C—O1C—Zn1—O1B	−177.71 (18)	O1B—Zn1—Zn2—O1A	118.88 (7)
Zn2—O1C—Zn1—O1B	39.37 (5)	O1C^i^—Zn1—Zn2—O1A	60.95 (7)
C2C—O1C—Zn1—Zn2^i^	−37.08 (19)	O1C—Zn1—Zn2—O1A	−119.05 (7)
Zn2—O1C—Zn1—Zn2^i^	180.0	O1A^i^—Zn1—Zn2—O1B	61.12 (7)
C2C—O1C—Zn1—Zn2	142.92 (19)	O1A—Zn1—Zn2—O1B	−118.88 (7)
C4A—O2A—Zn2—O2B	160.19 (16)	O1B^i^—Zn1—Zn2—O1B	180.0
C4A—O2A—Zn2—O2C	−109.27 (17)	O1C^i^—Zn1—Zn2—O1B	−57.93 (7)
C4A—O2A—Zn2—O1C	−22.6 (3)	O1C—Zn1—Zn2—O1B	122.07 (7)

Symmetry code: (i) −*x*, −*y*+1, −*z*.
